# Dr. Marshall Hall

**Published:** 1853-11

**Authors:** 


*Dr. Marshall Hall.*—The visit of this distinguished physiologist to this country, must needs have been a pleasant one to him, as well as profitable to those whose good fortune has permitted them to meet him. Urbane and modest in his manner, and indefatigably devoted to physiological research, he was everywhere met with a cordial welcome, and has everywhere made that welcome a labor of love to his entertainers, by the freedom with which he has imparted information as to his specialty.

Few men, perhaps no man, occupies so enviable a position in medical science, as Marshall Hall. In his elevation to eminence he has risen upon his own merits, and not at the expense of others. He has lived long enough to have attained and enjoyed a reputation as wide as the scientific world itself, and this without resort to any means discreditable to himself.

His fame had filled his native land. The tribute of praise from England and from continental Europe, had become familiar to him, and its novelty lost by repetition; when now in his old age he crosses an ocean, and visits a foreign country, to find his name a household word, and the kind offices of scientific and personal friendship everywhere extended to him by those of whose existence he scarcely knew.

It is a pleasure to us to recall the evening when for the only time we saw him, and listened to his demonstration of his theory. His *physique* is not imposing. In height he is below the middle stature, and is somewhat full in figure, though not corpulent. His countenance is a mild one, and beaming with good feeling, while his manner of speaking of his own great discovery, is modest and unassuming, but still positive. His style is lucid, and, in conducting his experiments, he leads the observer on, from step to step, to a full and lucid understanding of their meaning. Like all other discoverers, his own ideas of his subject are very clear, and he comes at once to his conclusions by the same process through which he first arrived at them. In this, detail is sacrificed, but the salient points of his theory stand prominently out, and are more easily grasped by the listener than if incumbered by minutiæ.

Dr. Hall is now in New York, and we were glad to notice that his presence graced the opening of the session at the College of Physicians and Surgeons, and that he still continues to teach the physiology of the nervous system. We understand that it is his intention to visit New Orleans, and witness there the vivisections of the alligator, which make that city a physiological center. He will find there men of talent and high scientific attainment to join in his investigations, and we may hope for interesting experiments and profitable results. S. B. H.

